# Childhood sexual abuse in pediatric-onset anorexia Nervosa: a systematic review

**DOI:** 10.3389/fped.2026.1886855

**Published:** 2026-09-03

**Authors:** Elena Bozzola, Flavia Cirillo, Giulia Spina, Maria Rosaria Marchili

**Affiliations:** Bambino Gesù Children’s Hospital, IRCCS, Rome, Italy

**Keywords:** anorexia nervosa, childhood sexual abuse, children, pediatric, trauma

## Abstract

**Background:**

Childhood sexual abuse (CSA) is a pervasive and underreported form of trauma strongly associated with psychiatric morbidity. While numerous studies in adult populations have demonstrated a significant association between trauma exposure and eating disorders, particularly anorexia nervosa (AN), limited evidence exists regarding this relationship in the pediatric population. Understanding how CSA relates to early-onset AN is essential for improving diagnostic accuracy and guiding trauma-informed treatment. Aim of the study was to explore the relationship between CSA and the onset of anorexia nervosa in individuals under 18 years of age, providing a more comprehensive assessment of trauma exposure as a contributing factor to AN development.

**Methods:**

A systematic review was conducted in accordance with PRISMA guidelines. PubMed and Embase were searched for studies including empirical data, participants under age 18, and assessed sexual abuse in relation to AN. Data were extracted from eligible studies and synthesized narratively.

**Results:**

Across multiple clinical and questionnaire-based studies, adolescents with AN exhibited notably higher rates of CSA compared to peers without eating disorders. CSA was associated with depressive symptoms, emotional dysregulation, impaired self-esteem, fear of life, and relational difficulties. Evidence also indicated subtype differences, with binge-eating/purging AN showing stronger association with CSA than restrictive AN. The relationship between CSA and AN appears mediated by emotional and interpersonal factors rather than being directly causal.

**Conclusions:**

CSA contributes significantly to psychological vulnerability in adolescents with AN. Trauma exposure influences mood, relational functioning, and coping mechanisms, all of which shape AN onset and severity. Systematic trauma assessment should be integrated into pediatric eating disorder evaluations, and treatment should incorporate trauma-informed and emotion-focused interventions to support holistic recovery.

## Introduction

Childhood sexual abuse (CSA) refers to the involvement of a minor in sexual activities perpetrated for the gratification or benefit of an adult or older adolescent. This includes molestation, statutory rape, sexual exploitation, child pornography, prostitution, and incest, all of which compromise the child’s physical and psychological safety (1). Global estimates indicate that CSA affects approximately 20% of females and 5%–10% of males, although these figures likely underestimate true prevalence because of underreporting related to concealment, fear, self-blame, and family denial (1, 2).

Extensive research in adult populations has established that childhood trauma—including sexual, physical, and emotional abuse—is significantly associated with eating disorders, particularly anorexia nervosa (AN). Individuals with CSA exhibit more severe eating disorder psychopathology, greater psychiatric comorbidity, lower self-esteem, and greater interpersonal difficulties (3)*.* CSA also disrupts emotional regulation and contributes to bodily dissatisfaction, which may serve as mechanisms linking trauma to disordered eating behaviours (4)*.*

Despite robust evidence in adults, comparatively little is known about how CSA influences the onset and progression of AN during childhood and adolescence. Moreover, available pediatric studies show considerable variability in their findings, with methodological differences, small sample sizes, and likely underreporting of abuse contributing to inconsistent results. Pediatric-onset AN presents unique developmental challenges, as early trauma may disrupt emotional development and body image formation. Furthermore, anorexia nervosa represents a major public health concern, as it is associated with substantial functional impairment, long-term medical complications, and the highest mortality rate among psychiatric disorders, underscoring the importance of early identification and intervention (5, 6).

Some authors have proposed that CSA acts as a mediator rather than a direct cause of AN, shaping vulnerability and symptom expression through complex psychological and interpersonal pathways (2).

Given the clinical significance of both CSA and AN during adolescence, a systematic review of pediatric populations is warranted to clarify the relationship between trauma exposure and early-onset eating disorders. A better understanding of these pathways may enable clinicians to implement comprehensive trauma assessments, adopt integrated therapeutic approaches, and support recovery trajectories that address both trauma histories and eating disorder pathology.

The aim of this work is to evaluate existing evidence on the relationship between childhood sexual abuse and the onset of anorexia nervosa in individuals younger than 18 years, with the goal of informing clinical practice and guiding future research.

## Material and methods

### Design of the study

This systematic review was conducted in accordance with PRISMA guidelines.

### Eligibility criteria

Studies were included if they met the following criteria:

− population: children and adolescents aged under 18 years
− exposure: documented history of CSA
− outcome: diagnosis of AN or evaluation of AN psychopathology.

Study Design: Research studies (e.g., observational, cross-sectional, case–control, or cohort designs) providing original data.

Studies were excluded if they were non-original articles, included exclusively adult populations or mixed samples without separable pediatric data, lacked sufficient methodological detail or empirical data relevant to the research question.

### Information sources and search strategy

#### A comprehensive literature search was executed across pubMed and embase

PubMed was searched using the Boolean string [“Anorexia Nervosa”(Mesh)] AND [“Child Abuse, Sexual”(Mesh)], applying the filters Humans and Child: birth–18 years. A secondary search was conducted in Embase using the following strategy: (‘anorexia nervosa'/exp OR ‘anorexia nervosa’) AND (‘child sexual abuse'/exp OR ‘child sexual abuse’) AND ([newborn]/lim OR [infant]/lim OR [child]/lim OR [preschool]/lim OR [school]/lim OR [adolescent]/lim) AND [humans]/lim.

Additional records were identified through manual reference checking of relevant literature.

#### Study selection

After removal of duplicate records, titles and abstracts were screened independently by two reviewers to identify studies potentially meeting the eligibility criteria. Records considered potentially relevant were retrieved in full text and independently assessed by the same reviewers against the predefined inclusion and exclusion criteria. Studies that did not meet the eligibility criteria were excluded, and the reasons for exclusion at the full-text stage were recorded. Any disagreements between the reviewers during the screening or eligibility assessment were resolved through discussion and consensus.

#### Data extraction and risk of bias

A standardized data extraction form was used to collect the following variables: first author, year of publication, country, study design, age profile of participants, sample size, and main findings. Methodological quality and risk of bias were assessed independently by two reviewers, with disagreements settled by consensus.

#### Data synthesis

A narrative synthesis was performed to summarize and integrate the findings of the included studies.

## Results

### Study selection

**Figure 1:**
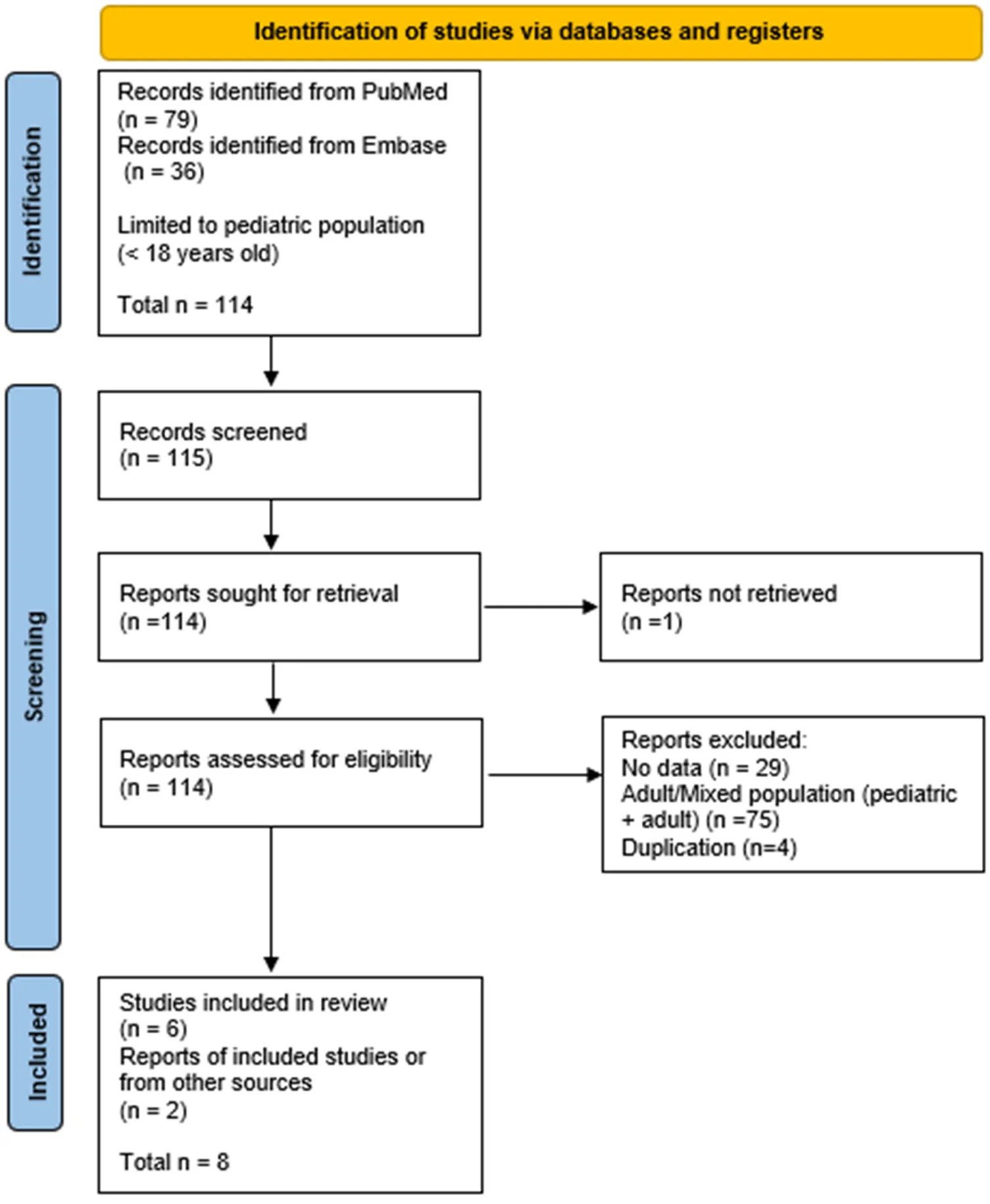
PRISMA flow diagram showing the identification, screening, eligibility assessment, and inclusion of studies investigating the association between childhood sexual abuse and anorexia nervosa in individuals younger than 18 years.

As shown in Figure 1, a total of 79 records were initially identified through the PubMed search strategy, of which six met the inclusion criteria after abstract screening and full-text review. The secondary Embase search yielded 36 records, and four of these were initially eligible; however, all were duplicates of those already identified in the primary search and were therefore removed. Two additional studies were identified through cross-referencing, resulting in a final total of eight studies included in the synthesis (7–14).

### Characteristic of included studies

All selected studies investigated the relationship between CSA and the onset or clinical presentation of AN in adolescents under 18 years of age (7–14).. Extracted data are presented in Table 1.

**Table 1:** Characteristics and principal findings of studies investigating childhood sexual abuse and anorexia nervosa in children and adolescents.

Reference	Study design	Country	Age reference (years)	Number of patients	Findings
Hicks White, 2018 (7)	Clinical study	USA	15.0 ± 1.7	182	Significant association between depressive symptoms and sexual abuse (*p* = 0.024).
Monteleone, 2023 (8)	Questionnaire	Italy	15.94 ± 1.28	354	Sexual abuse linked to reduced life satisfaction through emotional abuse and neglect; indirect effect on eating disorder mediated by emotional and relational factors.
Jaite, 2012 (9)	Questionnaire + Clinical interview	Germany	15.8 ± 1.6	121	Higher rate of sexual abuse in patients with anorexia nervosa binge-eating/purging anorexia than in restrictive anorexia
Stein, 2013 (10)	Clinical interview	Israel	16.2 ± 1.3	96	Fear of life associated with depression, body-related problems, and childhood sexual abuse.
Hölling, 2007 (11)	Questionnaire	Germany	11-17	7498	A significant association has been found with sexual harassment in patients aged 14–17 years
Jaite, 2013 (12)	Case–control clinical study	Germany	12–18	147	Adolescents with AN reported significantly higher rates of emotional, physical, and sexual abuse than psychiatric and healthy controls; abuse associated with greater psychopathological burden.
Gander, 2018 (13)	Clinical study + interview	Austria	13–18	120	Adolescents with AN showed higher attachment insecurity and significantly more childhood abuse and neglect than those with major depression; abuse related to disorganized attachment.
Spiegel, 2022 (14)	Clinical study	Germany	13–18	128	Emotional abuse significantly interacted with borderline personality traits in binge–purging eating disorder, intensifying symptom severity and impulsivity.

Legend: AN, anorexia nervosa.

### Association between CSA and AN

Studies reported a significant association between depressive symptoms and sexual abuse in adolescents undergoing eating disorder treatment (7, 10, 12). Adolescents with AN reported significantly higher rates of emotional, physical, and sexual abuse compared to psychiatric and healthy comparison groups (12). Abuse exposure was associated with higher overall psychopathological burden, depressive symptomatology, anxiety, and poorer global functioning (12). In subtype comparisons, Jaite et al. found higher rates of sexual abuse in the binge-eating/purging AN subtype compared to the restrictive subtype (9). Furthermore, analyzing a large youth sample, Hölling et al. identified a significant association between experiences of sexual harassment and the 14–17 year age group, supporting the relevance of sexual victimization during mid-adolescence as a factor contributing to eating pathology (11).

### Psychological and interpersonal correlates

The link between CSA and AN is complex and mediated by psychological and relational factors:

− attachment and relational distortions: abuse severity was strongly associated with attachment disorganization, with adolescents with AN showing significantly higher levels of attachment insecurity, disorganized attachment patterns, and higher frequencies of childhood abuse and neglect than depressive controls (13);
− mediating pathways: Monteleone et al. demonstrated that the relationship between sexual abuse and eating pathology was indirect and mediated by emotional abuse, neglect, and relational difficulties, which negatively impacted life satisfaction (8);
− personality interactions: Spiegel et al. identified that emotional abuse interacted with borderline personality traits to amplify eating disorder symptom severity and impulsivity in binge–purging presentations (14);
− affective dysregulation: fear of life was significantly associated with depression, body-related problems, and childhood sexual abuse, supporting the view that CSA acts as a vulnerability factor compromising emotional regulation and self-esteem (8, 10)

### Narrative synthesis

Across all studies, CSA was consistently associated with mood dysregulation, lowered self-esteem, impaired interpersonal functioning, and more severe AN symptoms in adolescents. These findings reinforce the need for systematic trauma screening within pediatric eating disorder assessments (3).

## Discussion

This systematic review highlights a clinically meaningful relationship between CSA and AN onset during the pediatric age, emphasizing the role of trauma as a significant vulnerability factor rather than a direct etiological trigger. CSA should be distinguished from the broader concept of child abuse (1).. CSA refers specifically to the involvement of a child in sexual activity that he or she cannot fully understand, cannot consent to, or for which the child is developmentally unprepared, and is characterized by profound violations of bodily integrity, trust, and personal boundaries (1). While all forms of child maltreatment have been associated with eating disorders, CSA appears to exert particularly strong effects on body image, shame, dissociation, interpersonal functioning, and emotional regulation, thereby increasing vulnerability to anorexia nervosa and other eating disorders (3, 15, 16). Childhood abuse is consistently associated with depressive symptoms, emotional dysregulation, relational impairment, attachment insecurity, and increased eating disorder severity (7–11). Beyond depressive symptoms, adolescents with anorexia nervosa and a history of CSA frequently present with multiple psychiatric comorbidities that may influence prognosis and treatment response. These include anxiety disorders, post-traumatic stress disorder, dissociative symptoms, obsessive-compulsive symptoms, self-harming behaviours, borderline personality traits, substance misuse in older adolescents, and increased suicidal ideation. Trauma-related emotional dysregulation and attachment insecurity may further complicate treatment adherence and increase the likelihood of chronic illness. Subtype analyses revealed that binge–purging presentations were especially linked to traumatic experiences (9). These findings are consistent with broader literature demonstrating that childhood maltreatment significantly increases the risk for eating disorder psychopathology across developmental stages (15, 16). Importantly, several studies demonstrated that the effect of sexual abuse on AN is largely indirect, mediated by emotional maltreatment, impaired self-esteem, attachment disturbances, and affective comorbidity (8). CSA is known to compromise emotional and psychological functioning through mechanisms such as shame, guilt, self-blame, dissociation, and impaired affect regulation (2). These psychological processes are supported by neurobiological models showing that early trauma produces long-term dysregulation of the hypothalamic–pituitary–adrenal axis and heightened limbic system reactivity, increasing vulnerability to maladaptive coping strategies such as restrictive eating and purging. Literature suggests that depressive symptoms frequently co-occur with CSA in adolescents with eating disorders, reinforcing evidence that depression may act as an important mediator between trauma exposure and restrictive eating patterns (7, 10). The convergence of CSA and AN during adolescence is particularly significant, as sexual victimization often peaks during the same developmental window in which AN most commonly emerges (7–9, 11). This temporal overlap suggests that CSA may function as a precipitating stressor during a period already characterized by profound biological, emotional, and identity-related changes. The relationship between CSA and eating disorders appears to be mediated through emotional abuse, neglect, and relational dysfunction, rather than acting independently (8). This finding aligns with comprehensive developmental psychopathology models in which early maltreatment alters attachment organization, self-worth, emotional regulation, and interpersonal sensitivity—core features implicated in the pathogenesis of AN (15). From this perspective, CSA contributes to a cumulative vulnerability process rather than serving as a single causal factor. A clinically important nuance emerges from subtype distinctions. Adolescents with binge-eating/purging AN show higher rate of sexual abuse compared to those with restrictive AN (9). This differentiation may reflect trauma-associated behavioural dysregulation, impulsivity, and affective instability, which can manifest through purging behaviours and loss of control over eating. These findings support the need for subtype-specific therapeutic approaches tailored to underlying trauma-related psychopathology. Taken together, the results support a conceptual model in which CSA contributes to anorexia nervosa onset in pediatric patients through a constellation of psychological and neurobiological effects including emotional dysregulation, depressive symptoms, body dissatisfaction, impaired relational functioning, and altered stress responsivity (3, 4, 16). From a clinical standpoint, the results of this systematic review confirm the crucial importance of integrating accurate and systematic assessment of traumatic experiences, including childhood sexual abuse, in all adolescents presenting with symptoms attributable to anorexia nervosa. Developmental AN cannot be fully understood without considering the contribution of traumatic factors, which—as demonstrated by the reviewed literature—can amplify psychological vulnerability and profoundly influence clinical presentation (3, 4, 15). Early identification of CSA allows clinicians to uncover hidden aspects of patients’ histories, often concealed due to shame, fear, or mistrust, and represents a prerequisite for designing targeted therapeutic interventions. A trauma-informed approach enables tailoring care to the patient’s specific emotional and relational needs, promoting emotional stabilization, improved therapeutic alliance, and greater nutritional compliance throughout treatment (16). Integrated interventions that address both eating disorder symptomatology and trauma-related psychopathology appear essential, particularly in youth with comorbid depression, dissociation, or attachment disturbances. Young people with AN who have a history of CSA represent a clinically fragile subgroup at high risk for psychiatric comorbidities, including major depression, emotion regulation deficits, dissociative symptoms, and suicidal ideation (10). Furthermore, given the high prevalence of underreported trauma, adolescents presenting with eating disorders should routinely undergo trauma-informed screening in a private, supportive, and non-judgmental setting after an appropriate therapeutic alliance has been established (17). The clinical interview should explore a history of sexual experiences or coercion, sexual harassment, childhood traumatic experiences affecting body image or eating behaviours, avoidance of discussing traumatic events, post-traumatic symptoms, self-harm or suicidal ideation. Positive findings should prompt a comprehensive psychological assessment, allowing the development of personalized, trauma-informed treatment integrated with nutritional rehabilitation and evidence-based psychological interventions. Large meta-analyses confirm that childhood sexual abuse significantly increases the risk of suicidal behaviour across the lifespan, underscoring the urgent need for careful risk assessment and safety planning in this population (18)..

This systematic review has several limitations. First, only eight studies met the inclusion criteria, limiting the strength and generalizability of the findings. Second, most of the available evidence originated from a restricted geographical area. Five of the eight included studies (62.5%) were conducted in German-speaking countries (Germany and Austria), while six studies (75%) originated from European populations. Consequently, the findings may not be fully representative of adolescents from other cultural, ethnic, or healthcare settings, where both the prevalence and disclosure of childhood sexual abuse and eating disorders may differ. Third, considerable heterogeneity existed among studies regarding methodology, trauma assessment instruments, sample characteristics, and definitions of sexual abuse, precluding quantitative synthesis. Finally, the retrospective assessment of traumatic experiences may have been affected by recall bias and underreporting, particularly considering the sensitive nature of CSA.

## Conclusion

These findings underline the importance of future research aimed at clarifying the mechanisms through which CSA influences the onset of AN in childhood and adolescence, with particular attention to interpersonal, familial, neurobiological, and temperamental variables. A deeper understanding of these pathways may facilitate the development of more effective prevention strategies, sensitive screening protocols, and increasingly personalized, trauma-informed therapeutic interventions.

## Data Availability

The original contributions presented in the study are included in the article/Supplementary Material, further inquiries can be directed to the corresponding author.
